# A Forecast Model for COVID-19 Spread Trends Using Blog and GPS Data from Smartphones

**DOI:** 10.3390/e27070686

**Published:** 2025-06-26

**Authors:** Ryosuke Susuta, Kenta Yamada, Hideki Takayasu, Misako Takayasu

**Affiliations:** 1 School of Computing, Institute of Science Tokyo, 4259 Nagatsuta-cho, Midori-ku, Yokohama 226-8502, Kanagawa, Japan; 2 Faculty of Global and Regional Studies, University of the Ryukyus, 1 Senbaru, Nishihara 903-0213, Okinawa, Japan

**Keywords:** COVID-19, social media data, GPS data, trend decomposition, robust variable selection, regression, forecasting

## Abstract

This study investigates the feasibility of using GPS data and frequency of COVID-19-related blog words to forecast new infection trends through a linear regression analysis. By employing time series’ trend decomposition and Spearman’s rank correlation, we identify and select a set of significant variables from the GPS and blog data to construct two models: a fixed-period model and a sequential adaptive model that updates with each new wave of infections. Our findings reveal that the adaptive model more effectively captures long-term trends, achieving approximately 90% accuracy in forecasting infection rates seven days in advance. Despite challenges in forecasting exact values, this research demonstrates that combining GPS and blog data through a dynamic, wave-based learning model offers a promising direction for enhancing the forecasting accuracy of COVID-19 spread. This approach has significant implications for public health preparedness.

## 1. Introduction

The recent global emergence and spread of infectious diseases, notably the COVID-19 pandemic, has profoundly impacted public health, economies, and societal structures. This situation underscores the urgent need for accurate forecasts of infection trends and rapid implementation of effective countermeasures. Research efforts to forecast the spread of COVID-19 have explored diverse data sources and modelling approaches, from traditional models like the Susceptible–Infectious–Recovered (SIR) model [1] and its extension, the Susceptible–Exposed–Infectious–Removed (SEIR) model [2], to advanced deep learning techniques [3,4,5]. Additionally, time series analyses based on case reports have employed the Auto-Regressive Integrated Moving Average (ARIMA) model [6,7], its seasonal generalisation, the Seasonal ARIMA (SARIMA) model, and the decomposable additive Prophet model [8,9], demonstrating the variety of methodologies applied in understanding and forecasting the pandemic’s dynamics.

Amidst these methodologies, integrating real-time digital traces has become indispensable to contemporary epidemiology. Smartphone-GPS data, for instance, support fine-grained models of influenza diffusion and COVID-19 risk [10,11,12], while social-media streams enable sentiment analysis [13], tweet-level surveillance [14], and symptom-based hotspot forecasting [15]. Web-search behaviour adds a complementary lens: Parpoula et al. showed that Greek Google searches for mental health services dwindled during the 2008–2013 economic crisis and rebounded only after economic stabilisation [16], whereas Chu et al. constructed a multivariate search-volume index that anticipated long COVID prevalence and revealed a shift from somatic to psycho-social concerns as public awareness grew [17]. Although these signals are timely, they face well-known challenges—delayed effects, ambiguous user intent, and regional search-engine biases. Moreover, daily case counts remain highly sensitive to policy shocks and new variants such as Omicron, producing abrupt fluctuations that conventional models struggle to capture [8]. Together, these observations motivate our hybrid approach, which fuses semantically rich blog posts with high-resolution mobility data to enhance real-time infectious disease forecasting.

In exploring forecast models for infectious diseases, researchers have encountered significant challenges, including the nonstationarity of data, a vast pool of potential variables, and, notably, shifting correlations over time. These challenges are further compounded by fundamental statistical issues inherent to epidemiological surveillance data. Traditional Statistical Process Control (SPC) techniques, widely used in industrial quality control, cannot be directly applied to health care monitoring problems due to violations of core assumptions such as stationarity, known asymptotic distributions, and independence of observations [18]. Epidemiological time series exhibit complex seasonal patterns, multiple change-points corresponding to epidemic onsets and terminations, and heteroscedastic variance structures that violate standard regression assumptions. Furthermore, the baseline establishment process, which is a critical step in outbreak detection, often relies on arbitrary data exclusion rules (such as removing the highest 15–25% of observations) that lack statistical justification [19]. These methodological limitations highlight the need for distribution-free approaches that can handle nonstationary processes and detect multiple level shifts without requiring prior knowledge of the underlying probability distribution.

The case of Google Flu Trends exemplifies the importance of adapting models to evolving data and user behaviour. Initially showing high accuracy in forecasting influenza outbreaks, its performance declined over time, underscoring the necessity of updating models to maintain their forecasting power [20,21,22].

Addressing these challenges, our study introduces a novel approach to forecasting modelling by combining GPS data and the frequency of COVID-19-related words in blog posts. Given the combination of these data sources, there is a large number of candidate variables. Therefore, we perform a linear regression analysis after extracting important variables based on the variable-selection method proposed by Yamada et al., [23]. By employing a sequential learning approach that updates with each estimated infection wave, we aim to capture long-term trends in infection rates accurately. This methodology seeks to mitigate the limitations observed in static models and respond dynamically to changes in the data landscape.

This study is structured as follows: Section 2 outlines the data used in this study and describes the preprocessing methods, defining the dependent and independent variables. Section 3 details the variable-selection methods to extract variables highly correlated with the dependent variable. Section 4 compares the results of a linear regression model utilizing selected variables (a fixed-period model) with those from a sequential adaptive model, segmented by infection-wave window. Finally, Section 5 discusses the method and data’s features, advantages, challenges, and potential applications of this approach. Our main contributions are summarized as follows: (i) we integrate high-resolution mobility traces with semantically rich blog texts, two data sources that have rarely been jointly utilised in prior research; (ii) we introduce wave-wise sequential re-training that preserves predictive power under pronounced nonstationarity; and (iii) we lay the groundwork for a trend-decomposition–based Pearson correlation measure that enables variable selection on nonlinear, drifting series, the formal definition of which is presented in Section 3. In an era where diverse big data sources are increasingly available, the development of algorithms capable of efficiently identifying relevant predictors from a large pool of variables and adapting to nonstationary data has become ever more important. This structured approach offers valuable insights for enhancing public-health strategies and response planning in the face of evolving infectious diseases.

## 2. Data and Preprocessing

### 2.1. Number of New Infections

This study analysed the daily progression of new infection counts sourced from the open data of the Ministry of Health, Labour, and Welfare [24], designating these counts as the dependent variable. The raw data on new infection numbers revealed nonlinear characteristics, exhibiting periodicity and notable variances in mean and variance across different waves of infection, which necessitated suitable preprocessing.

**Figure 1:**
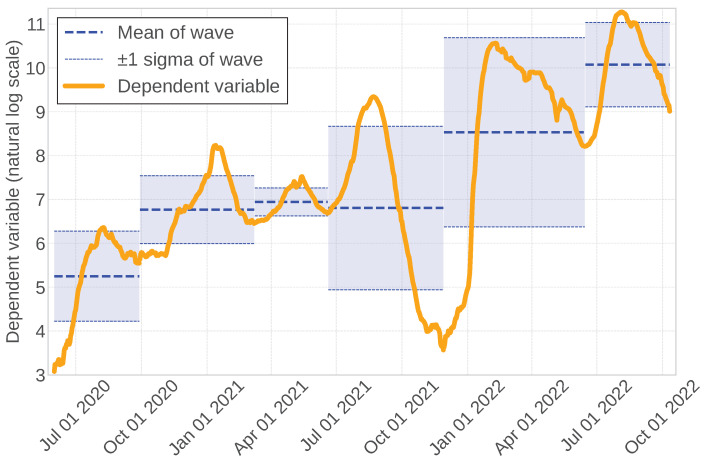
Time series of the dependent variable after pretreatment. Mean values (blue dotted line) and 1-crisis sigma (blue bandwidth) are illustrated for each period of each infected wave.

The reporting of new infection counts tends to be lower at the beginning of the week due to the closure of many medical institutions on Saturdays and Sundays. We smoothed the data to diminish this weekend effect by calculating the moving average over the preceding seven days. The magnitude of new infection counts fluctuates considerably over time; thus, we applied a natural logarithm transformation to preserve the original series’ trend and minimize variations in the data. Following these preprocessing steps, we set y(t) as in Equation (1) and show it in Figure 1, where x(t) is the number of new infections at time *t*.

(1) $$\begin{array}{c} y\left(t\right)=\ln\left(\sum_{k=0}^{6}x\left(t-k\right)\right) \end{array}$$

### 2.2. GPS Data

The GPS data for this study came from smartphone apps integrated with Agoop’s SDK, provided by Agoop Corp. Before their distribution, the data underwent cleansing, anonymization by privacy policies, and irreversible processing to estimate attributes and states.

Key data elements included daily IDs (refreshed at midnight to prevent tracking), timestamps, geographic coordinates, GPS accuracy (indicating a 68% likelihood for Android users to be within the accuracy radius, which was under 20 m for approximately half of the data points).

Following the methodology of previous research [12], the data purchased from Agoop were processed as follows: selection of users based on data transmission criteria, exclusion of iOS users due to uneven data transmission, and focusing solely on Android users.

Positional data were interpolated every 15 min using linear interpolation between the two closest data points or directly using matching timestamps. Home and work locations were estimated for each user within 1 km squares based on Agoop’s city code estimates but were refined for this study. After processing, data for about 400,000 users were summarized.

Time series data were created for four behavioural patterns (Home, Work, Move, Stay) at 15-min intervals, aggregating the number of people in each state across the target area. The probability of coming into close contact sufficient for infection was assumed to be proportional to the square of the number of people present in each 1 km^2^ area, for each behavioural pattern. Therefore, the square of the number of people was calculated to determine the risk of infection.

As the number of users targeted for data collection tended to decrease over time, normalization was performed using the following formula to correct for the person weight and eliminate the overall downward trend:

(2) $$\begin{array}{c} {\hat{z}}_{\mathrm{pattern}}\left(t\right)=\frac{S(0)}{S(t)}z_{\mathrm{pattern}}\left(t\right) \end{array}$$

Here, S(t) represents the total number at time *t*, and $z_{\mathrm{pattern}}\left(t\right)$ is the original series of the behavioural pattern time series. This normalization allowed us to observed fluctuations in the number of people going out over time. A similar normalization was applied to the squared time series using the squared normalization term from Equation (2).

**Figure 2:**
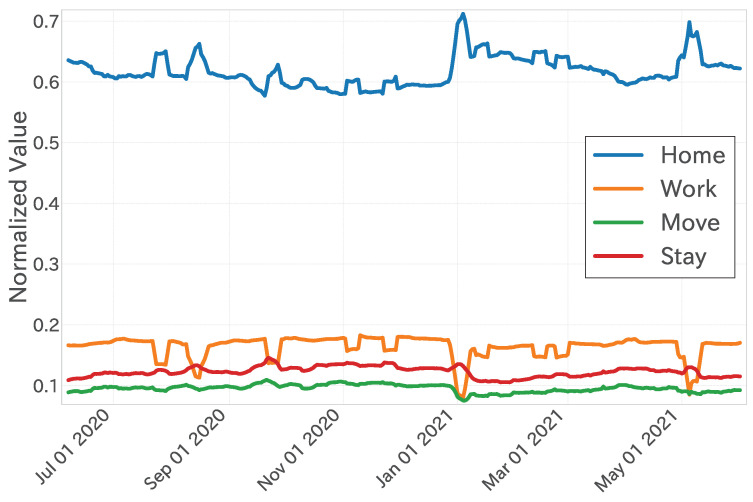
Behavioural pattern time series with normalization (Equation (2)) and moving average for the last 7 days in the context of the Greater Tokyo Area (including the prefectures of Tokyo, Saitama, Chiba, Kanagawa, Ibaraki, Tochigi, Gunma, and Yamanashi).

To facilitate the interpretation of the regression analysis results, the behavioural pattern time series was normalized to a range from 0 to 1 using Min-Max normalization after applying a 7-day moving average to smooth short-term fluctuations. The resulting time series of the four behavioural patterns (Home, Work, Move, Stay) is shown in Figure 2.

### 2.3. Blog Data

The blog data utilised in this study were gathered from Hotto Link Inc. Corporation’s kuchikomi @ kakaricho. This dataset encompasses a daily time series of frequencies for each word appearing alongside “コロナ” (COVID-19) in blog posts. The approach for selecting candidate words as independent variables unfolded as follows.

Beginning in June 2020, a daily sample of up to 1000 articles mentioning “コロナ” (COVID-19) underwent analysis, with dependency parsing conducted via the open-source Japanese NLP library GINZA, grounded in Universal Dependencies [25]. Using a dependency analysis, words directly related to “コロナ” (COVID-19) and those indirectly related through a second-level dependency relationship were identified. From this analysis, the top 600 words were compiled into a ranking and chosen as candidate independent variables. The top three words were “影響” (impact), “対策” (counter-measure), and “なる” (become).

Given the gradual decline in the number of blog data users, the frequency time series for each word was normalized against the total blog user count. These word-frequency time series were estimated daily and, similar to the new infection numbers and GPS data, were subject to short-term fluctuations, necessitating the application of a seven-day moving average.

The selection of candidate words for independent variables targeted those with direct or indirect connections to “コロナ” (COVID-19). However, due to sampling limitations, words of infrequent co-occurrence may also have been included. Upon gathering the frequency time series for words appearing with “コロナ” (COVID-19) from Kuchikomi@kakaricho, the average daily frequency during the study period was assessed, excluding words with minimal occurrences, such as “亡くなる” (die) or “ビジネスモデル” (business model). Additionally, words with an average daily posting frequency of less than 10 were removed from consideration.

Furthermore, words exhibiting abrupt frequency surges within specific periods and subsequently seldom appearing were omitted. This exclusion aimed to remove words with ephemeral correlations to the dependent variable that lacked consistency. Given the cyclic peaks in new infection counts, correlating words often displayed similar patterns. Words influenced by particular events, such as “オリンピック” (Olympics) or “給付金” (subsidy), that might compromise forecast stability were problematic.

To exclude peak words, we analysed power-law distributions characterizing peaks [26], where peak words typically demonstrated a significant disparity between the peak maximum and median. In parallel when removing low-frequency words, an examination excluded words whose peak-to-median ratio exceeded eight.

### 2.4. Creation of Lagged Time Series

This study’s time series data concerning the number of new infections, as discussed in Section 2.1, served as the dependent variable for constructing the forecasting model. For the independent variables, we utilised the lagged time series of GPS and blog data outlined in Section 2.2 and Section 2.3, respectively. Unlike traditional data analysis, a time series analysis necessitates incorporating not only present data but also historical information, thereby adding a layer of complexity to the study [27].

Significant correlations between data from two weeks earlier and the adequate reproduction number of new infections have been identified in previous research [12]. Studies leveraging the Baidu Index have observed trends of specific symptom-related searches peaked before the reported infection numbers [28]. Additionally, Cuihua and colleagues demonstrated that lagged data on symptom onset posts from Weibo enhanced regression model performance [29].

These time lags represent the timeline from COVID-19 infection to incubation, symptom onset, home isolation, seeking medical advice, undergoing testing, and eventually reporting. Given that this study incorporated 632 time series as potential independent variables, each with possible correlations to COVID-19 at varying time delays, we generated 21 lagged time series for each variable, ranging from 7 to 28 days. The subsequent section elaborates on the process to determine the optimal time delays. By leveraging these lagged time series, the model was designed to forecast the number of new infections with a minimum seven-day lead time.

## 3. Methodology

### 3.1. Flowchart

**Figure 3:**
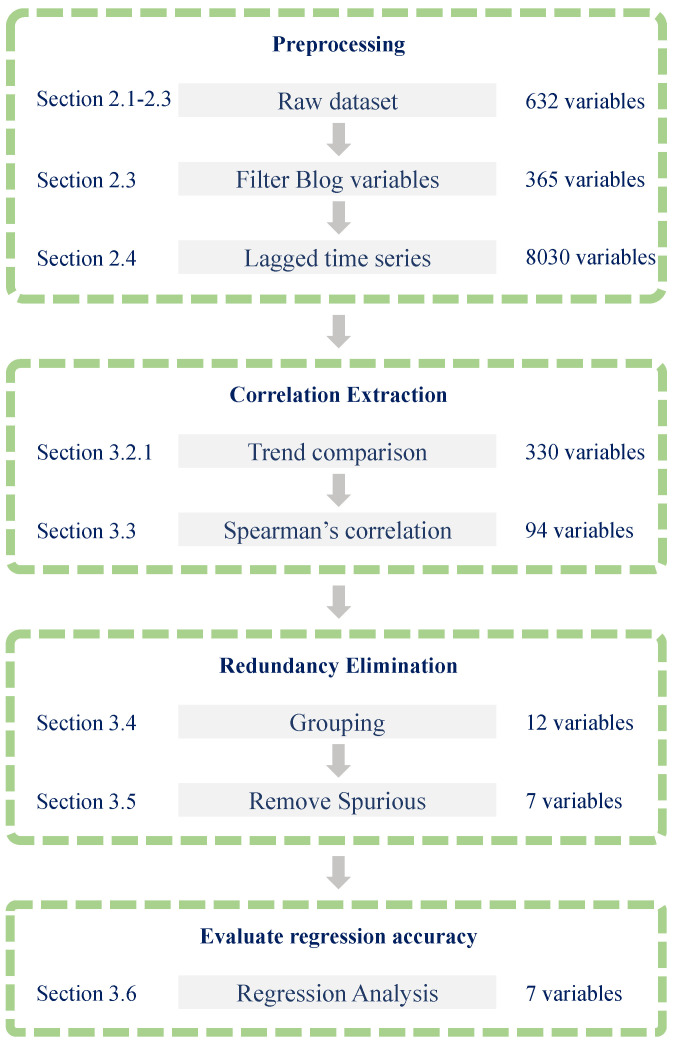
Flowchart of the variable selection process. The central grey area illustrates the sequential steps from raw data to preprocessing, correlation extraction, redundancy elimination, and regression accuracy evaluation. The left side indicates the corresponding sections of the paper for each step, while the right side presents the number of remaining candidate variables resulting from the variable selection process, as described in Section 4.1 (fixed-period model).

This study included 632 potential independent variables, consisting of 32 time series of behavioural patterns derived from GPS data and 600 time series of word frequencies obtained from blog data. Moreover, as elucidated in Section 2.4, considering time lags ranging from a minimum of 7 days to a maximum of 28 days, there were 13,904 lagged time series candidates for independent variables. Figure 3 depicts the approach for selecting variables through a flowchart.

The initial steps were dedicated to identifying independent variables that strongly correlated with the number of new COVID-19 infections. Subsequent steps focused on grouping among independent variables to mitigate multicollinearity and exclude variables with potential spurious correlations. The final selection of independent variables was based on comparing the adjusted coefficient of determination from the linear regression analysis with randomized time series.

### 3.2. Time Series’ Trend Correlation

Initially, this study characterized a trend through a binary representation of upward and downward trends, decomposed via the time series’ trend decomposition approach delineated below.

Sousa et al. [30] introduced a method for decomposing time series into upward and downward trends, employing the “epsilon–tau procedure” for financial datasets. We adapted that method for our analysis of dependent and independent variables.

**Figure 4:**
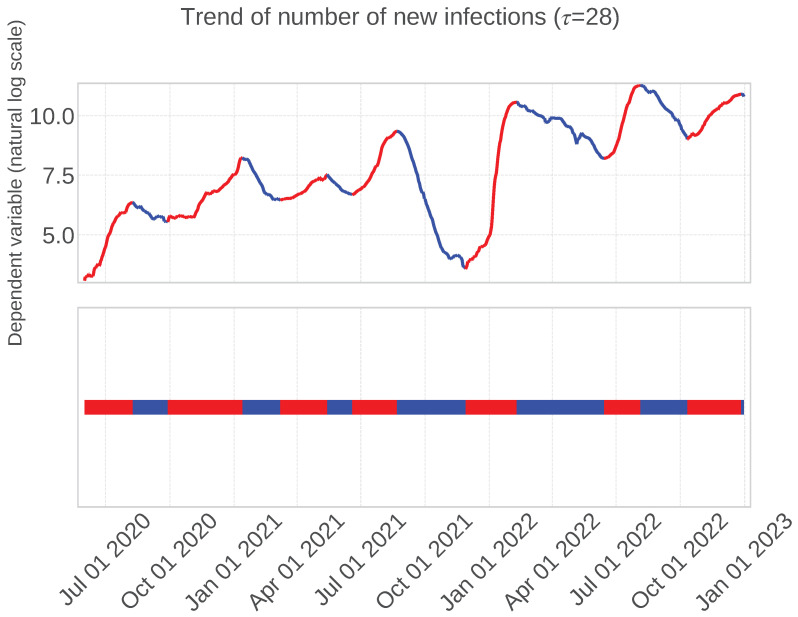
**Top**: Trend decomposition results (τ=28) of the dependent variable time series. **Bottom**: One-dimensional projection of trend decomposition, where red shows an up trend, and blue shows a down trend.

Figure 4 illustrates the implementation of the epsilon–tau procedure on the dependent variable. Here, the time constant parameter τ was fixed at 28, suitable for selecting the independent variables. A thorough examination of Figure 4 verifies that this methodology efficaciously segments the phases of infection waves despite transient fluctuations within each trend.

Moreover, as depicted in the lower segment of Figure 4, the outcomes of trend decomposition are manifested as a binary time series, assigning 0 (blue) for a downward trend and 1 (red) for an upward trend. This binary representation of trend time series laid the foundation for efficaciously capturing the prolonged trends of infection waves in the analysis presented in Section 3.2.1.

#### 3.2.1. Trend Series Pearson Correlation Coefficient

In this research, we introduce a method to assess the similarity between the trend series of the dependent variable and those of the independent variables. Firstly, we employed the epsilon–tau (ϵ–τ) procedure introduced by Sousa et al. [30], which suppresses short-lived fluctuations and labels each time point as either an upward or a downward segment once the run length exceeds the threshold τ. After decomposing the trends, we represented an upward trend by 1 and a downward trend by 0, thus generating a binary time series; this 0/1 encoding deliberately discarded amplitude differences and kept only the direction of movement. Next, we computed the Pearson product-moment correlation coefficient between the binary time series of the dependent variable and those of the independent variables.

The Pearson product-moment correlation for binary series mathematically is equivalent to the Matthews Correlation Coefficient (MCC), which is extensively utilised in binary classification tasks. The MCC yields high scores for models that accurately predict both positive and negative examples [31]. Because an infection dataset typically contains many more days of decline than of growth, this symmetry makes MCC less biased than metrics such as Recall, precision, or plain accuracy, which can over-reward the majority class. Boughorbel et al. [32] underscored MCC’s balance and robustness relative to AUC, accuracy, and F1-score through experiments using 10,000 randomly labelled class data. Therefore, in our analysis, the binary series’ Pearson product-moment correlation, congruent with the MCC outcomes, was a dependable metric for assessing the correlation of series data.

Employing the Pearson product-moment correlation for binary series after trend decomposition is vital for pinpointing the timing of shifts between infection phases in new-infection forecasting. Practically, detecting a switch from 0 (decline) to 1 (rise) even a few days in advance allows public-health officials to scale testing sites and hospital capacity before case counts visibly surge. Identifying infection-status transitions is crucial for the forecast process, underscoring our decision to utilise the trend series’ Pearson product-moment correlation for initial variable selection.

#### 3.2.2. Determination and Application of Threshold Using Otsu’s Method

Following the trend decomposition outcomes discussed in Section 3.2.1, this study incorporated Otsu’s method [33] to establish a threshold for the absolute Pearson product-moment correlation coefficients between the trend time series of the dependent and independent variables. Otsu’s method, originally developed for image binarization in greyscale and variance images, analyses the distribution of the absolute correlation coefficients to divide the dataset into two distinct categories.

**Figure 5:**
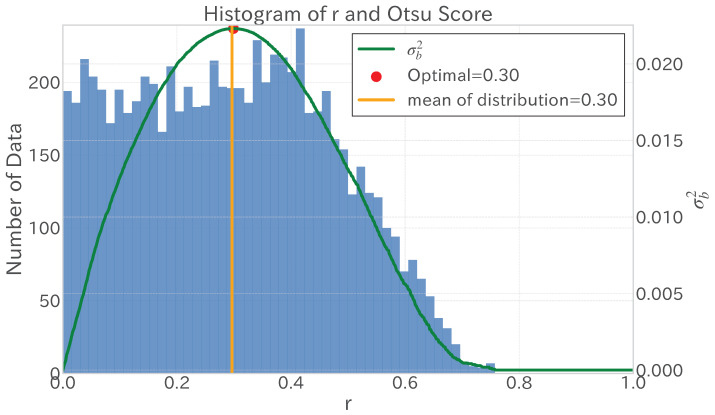
An example of applying Otsu’s method to the distribution of the absolute values of Pearson product-moment correlation coefficients for the trend time series analysed from 1 June 2020 to 31 May 2021. This figure illustrates the thresholding process (green line) that maximizes the between-class variance and the distribution (blue histogram) for data points within the range [0, 1]. The optimal threshold point (red) is identified and, as shown, tends to take a value close to the mean of the distribution (yellow line) in the absence of a clear bimodal distribution.

When observing the distribution of absolute Pearson product-moment correlation coefficients, the method identifies segments of weak and strong correlations. Otsu’s method then automatically determines the threshold by setting the division point. However, distributions might not always clearly show this two-part nature. As depicted in Figure 5, distributions frequently mimic a uniform pattern; however, Otsu’s method still allows for threshold establishment near the distribution’s mean.

The key benefit of Otsu’s method is its nonparametric essence, which does not assume anything specific about the data distribution. This characteristic is especially advantageous for determining an apt threshold regardless of the correlation coefficients’ distribution shape, offering a pragmatic approach even when distributions approximate a uniform profile. Integrating various variable selection strategies, a moderate threshold around the distribution’s mean effectively filters independent variables that are relevant to the dependent variable in this study’s framework.

#### 3.2.3. Determination of Optimal Lag Days for Independent Variables

As outlined in Section 2.4, this study treated all independent variables as lagged time series, because the correlation between public activities and the number of COVID-19 cases might not be the strongest at the same time point. By applying the Pearson product-moment correlation to the trend time series, we identified the optimal lag days where the absolute value of the correlation coefficient between each independent variable and the dependent variable was maximized. The optimal lag day for each variable was determined as follows:

(3) $$\begin{array}{c} \hat{k}=\arg\max_{k}\left|\mathrm{corr}(X_{i}\left(t-k\right),y\left(t\right))\right| \end{array}$$

where $\hat{k}$ is the lag day that maximizes the absolute value of the Pearson correlation coefficient between the lagged independent variable $X_{i}\left(t-k\right)$ and the dependent variable y(t).

This methodology enabled the assessment of the correlation strength between each independent variable and the dependent variable, pinpointing the optimal number of lag days where the correlation between the independent and dependent variables was the strongest. This was particularly relevant for variables likely to have a temporal causal relationship with the dependent variable, as accurately determining the optimal lag days significantly enhanced the forecasting model’s accuracy.

For example, analysing the trends in infectious disease spread allowed us to identify the temporal precedence of public movements or social media activities, as reflected in behavioural pattern time series or the volume of blog posts. This analysis can clarify how changes in public behaviour or online engagement precede changes in new infection counts, providing crucial insights for forecasting and managing infectious disease outbreaks.

### 3.3. Variable Selection Using Spearman’s Rank Correlation

Pearson correlation of trend time series is good at identifying variables that have a linear relationship with the trend. However, it may fail to detect dynamics associated with sudden increases or decreases within a time series. Such rapid changes are very important because they may indicate abrupt changes in underlying phenomena that have a significant impact on the dependent variable, which are often overlooked in trend analysis. To capture these important dynamics, we employed a Pearson analysis of trend time series followed by Spearman’s rank correlation.

In this investigation, Spearman’s rank correlation [34] was utilised to refine the selection of variables previously identified through the Pearson product-moment correlation of trend time series. These variables, which showed significant correlations with the dependent variable, enhanced the forecasting model’s accuracy. Spearman’s rank correlation, a nonparametric measure, evaluates correlations based on data ranks, emphasizing the ordinal relationships among data points without the influence of the actual data values. This feature proves advantageous for analysing data exhibiting nonlinear relationships. Furthermore, this method has been successfully applied in the field of infectious diseases, where it has proven effective for analysing complex, nonlinear interactions between various factors [35].

The process involved calculating the absolute values of Spearman’s rank correlation coefficients between the dependent variable and each selected independent variable’s time series. After this step, Otsu’s method established a threshold for determining correlated independent variables, as detailed in Section 3.2.2.

The rationale for selecting Spearman’s rank correlation derived from its robustness against nonstationarity and outliers within the dependent variable’s dataset. The dependent variable displayed periodic patterns corresponding to each infection wave alongside observable long-term trends, indicating that the data deviated from a normal distribution. Similar characteristics were noted in the independent variables. Consequently, Spearman’s rank correlation was preferred for its ability to uncover genuine correlations unaffected by the data’s nonlinear nature or outliers.

### 3.4. Grouping of Independent Variables

The selection process identified independent variables that demonstrated correlations in trends or rankings over time with the dependent variable. Numerous pairs or groups were expected to exhibit strong correlations among these variables, especially when their time series were highly correlated. For example, time series that represent word frequencies of related terms or behavioural patterns estimated from GPS data may show high inter-correlations. Such scenarios give rise to the issue of multicollinearity.

Multicollinearity inflates the standard errors of the regression coefficients, increasing the likelihood that significant independent variables are erroneously judged as statistically insignificant [36]. This outcome hampers the model’s interpretability and may adversely affect its capability to accurately predict future values of the dependent variable. The model’s stability is also at risk, as the regression coefficients could exhibit substantial variability by including or excluding independent variables.

To address the issue of multicollinearity, this research employed Spearman’s rank correlation for grouping highly correlated independent variables, selecting a representative variable from each group based on the highest absolute value of Spearman’s rank correlation coefficient with the dependent variable. This strategy aimed to improve the model’s interpretability and forecasting precision.

### 3.5. Removal of Spurious Correlations

When identifying variables with strong correlations with the dependent variable and grouping time series with similar fluctuation patterns, not all selected independent variables necessarily maintain a genuine causal relationship with the dependent variable. This suggests spurious correlations, which may arise due to coincidental alignment with other variables. To address this issue, probabilities derived from Bayesian estimation were employed to discern and eliminate such spurious correlations [23].

Here, P(A:B) quantifies the measure of the influence of variable *A* on the target variable when the condition of variable *B* is given. The lower the value of P(A:B), the more variable *B* explains the association between variable *A* and the target variable, suggesting that the correlation between variable *A* and the target variable may be spurious.

To assess the asymmetry in the influence between variables, the following metric was defined:

(4) $$\begin{array}{c} D\left(A:B\right)=\frac{P(A:B)-P(B:A)}{P(A:B)+P(B:A)}. \end{array}$$

This metric evaluates the presence of spurious correlation of variable *A* due to variable *B* using P(A:B) and D(A:B).

### 3.6. Performance Evaluation Method for Variable Selection in Regression Analysis

Consider a linear regression model where we incorporate lagged values of the independent variables to predict the dependent variable:

(5) $$\begin{array}{c} y_{t}=\beta_{0}+\beta_{1}X_{1,t-k_{1}}+\beta_{2}X_{2,t-k_{2}}+\ldots +\beta_{p}X_{p,t-k_{p}}+\epsilon_{t} \end{array}$$

where $y_{t}$ is the dependent variable at time *t*, $X_{i,t-k_{i}}$ represents the *i*th independent variable lagged by $k_{i}$ time steps, $\beta_{i}$ are the coefficients to be estimated, and $\epsilon_{t}$ is the error term at time *t*.

In a linear regression analysis, the choice of variables is crucial to model accuracy and robustness. Particularly when dealing with time series data, it is essential to address issues like overfitting.

We present a methodology for finalizing variable selection in regression analysis, inspired by the approach proposed by Yamada et al. [23]. This methodology involves a few steps. Firstly, we calculate the difference time series for the chosen independent variables. Then, we shuffle this series to randomize its fluctuations. Next, we generate a random walk time series from the shuffled series. We then use this random walk time series for a linear regression analysis to fit the dependent variable. Finally, we calculate the adjusted coefficient of determination, denoted by ${\tilde{R}}^{2}$, to assess the effectiveness of this fitting.

(6) $$\begin{array}{c} {\tilde{R}}^{2}=1-\frac{\sum_{i=1}^{n}{\left(y_{i}-{\hat{y}}_{i}\right)}^{2}/\left(n-p-1\right)}{\sum_{i=1}^{n}{\left(y_{i}-\bar{y}\right)}^{2}/\left(n-1\right)} \end{array}$$

where $y_{i}$ denotes the observed values, ${\hat{y}}_{i}$ the predicted values, $\bar{y}$ the mean of observed values, *n* the sample size, and *p* the number of model parameters.

The methodology for selecting independent variables begins by ranking these variables based on the absolute values of their correlations with the dependent variable, calculated using Spearman’s rank correlation. This approach marks a departure from traditional methods which may use different indices.

Once variables are ranked, the analysis commences with the highest-ranked independent variable. For each configuration of variables, the adjusted coefficient of determination, ${\tilde{R}}^{2}$, is calculated to evaluate the fit of the linear regression model.

To ensure the robustness of the ${\tilde{R}}^{2}$ values obtained, 10,000 simulations were performed with the variables randomized within the time series. This process generated a distribution of ${\tilde{R}}^{2}$ values from which the upper 5% was used as a significance threshold. This threshold helped in determining the statistical robustness of the ${\tilde{R}}^{2}$ achieved with the original data.

The process of adding variables to the model was iterative:

- Begin with the model that includes the top-ranked variable.
- Add one variable at a time, recalculating ${\tilde{R}}^{2}$ for each new model.
- Continue adding variables until the ${\tilde{R}}^{2}$ of the model exceeds the significance threshold determined from the simulations.

The optimal number of variables to include in the model was determined using the following criterion:

$$\mathrm{Optimal}\;\mathrm{Variables}=\arg\max_{k}\left\{k:{\tilde{R}}_{k}^{2}\geq \mathrm{Threshold}\right\}$$

where ${\tilde{R}}_{k}^{2}$ is the adjusted coefficient of determination for the model using the top *k* ranked variables. This ensured that the model included the smallest number of variables needed to achieve a ${\tilde{R}}^{2}$ that surpassed the established threshold, thereby maximizing explanatory power while minimizing the risk of overfitting.

**Figure 6:**
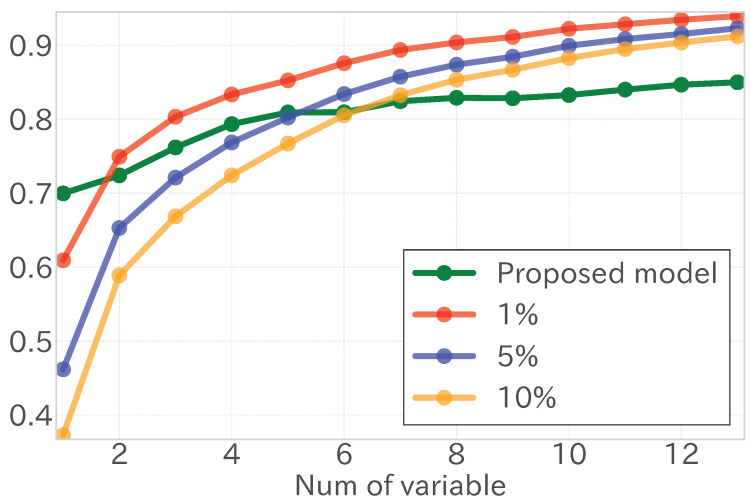
An illustrative graph showing the relationship between the number of independent variables (horizontal axis) and the adjusted coefficient of determination (vertical axis) for the original time series of independent variables (green) and the top 1% (red), 5% (blue), and 10% (yellow) of the random walk time series.

If the ${\tilde{R}}^{2}$ from the model with fewer variables consistently fell short of the threshold, the variable count that first exceeded the top 5% of the randomized results was selected. If this criterion was not met, the selection focused on maximizing the ${\tilde{R}}^{2}$ for the original series, as depicted in Figure 6.

This strategy facilitated the automatic identification of the most practical combination of independent variables while affirmatively establishing the proposed method’s statistical advantage over the random walk model.

### 3.7. Forecast-Model Specification

Building on the preprocessed series (Section 2) and the variable-selection pipeline (Section 3), we considered two linear-regression frameworks that differed only in the definition of the learning window.

#### 3.7.1. Fixed-Period Model

The training set comprised all observations from 1 June 2020 to 31 May 2021, while the evaluation was performed on 1 June 2021–12 October 2022. Let $y_{t}$ denote the 7-day-smoothed log count of new infections on day *t* and ${\left\{x_{i,t-k_{i}^{*}}\right\}}_{i=1}^{p}$ the *p* lagged independent series selected in Section 3, each shifted by its optimal lag $k_{i}^{*}\in \left\{7,\ldots ,28\right\}$. Then, the model is

(7) $$\begin{array}{c} y_{t}=\beta_{0}+\sum_{i=1}^{p}\beta_{i}\;x_{i,t-k_{i}^{*}}+\varepsilon_{t},\;\varepsilon_{t}∼N\left(0,\sigma^{2}\right) \end{array}$$

and parameters ${\left\{\beta_{i}\right\}}_{i=0}^{p}$ are estimated by ordinary least squares (OLS). The minimum lag of 7 days guarantees that the earliest forecast horizon is one week.

#### 3.7.2. Sequential Adaptive Model

To adapt the pronounced nonstationarity of pandemic dynamics, we segmented the timeline into infection-wave windows via the ϵ–τ trend decomposition (Section 3.2; τ=28). In this study, the wave boundaries were determined ex post from the complete time series so that the evaluation could use the ground-truth windows; for wave *w* (w≥3), we defined

$${\mathrm{train}}_{w}=\left\{\mathrm{waves}\;w-2,w-1\right\},\;{\mathrm{validate}}_{w}=\left\{\;\mathrm{wave}\;w\;\right\}.$$

Within each confirmed ${\mathrm{train}}_{w}$ block, we (i) recomputed trend correlation and Spearman correlation, (ii) re-applied grouping and spurious removal, and (iii) re-estimated the regression coefficients by OLS. Thus, both the active feature set $X_{w}$ and the coefficient vector $\beta_{w}$ were updated at every wave, enabling the model to track shifting word usage and mobility patterns.

For real-time operation, where the boundary of an emerging wave cannot be known in advance, the system continues to issue forecasts with the model trained on the two most recently completed waves until τ consecutive days of monotonic growth or decline accumulate. Once this τ-day criterion is met, the boundary is locked, and the sequential model is retrained with the newly closed wave pair, mirroring the retrospective procedure described above.

Forecasts from successive validation windows were concatenated to form a series aligned with the fixed-period test span, permitting direct comparison with a fixed-period model.

### 3.8. Evaluation Method for Forecast Results

We applied the dataset and variable selection methodologies delineated in Section 2 and Section 3 to estimate and predict the dependent variable using linear regression models. To assess the model’s performance, we employed the following metrics.

Initially, the mean absolute error (MAE) was utilised to evaluate the performance of linear regression models. The MAE offers a straightforward measure of the model’s forecast error magnitude by averaging the absolute differences between observed and predicted values. This is especially relevant when analysing regression outcomes using multiple variables. As elaborated in Section 4.2, it was necessary to account for variations in data mean and variance across different periods. Therefore, the MAE was considered an appropriate metric for comparing models’ overall performance across various data intervals.

Furthermore, we introduced an indicator to assess the model’s ability to capture long-term trends in observed values. Employing the trend decomposition method highlighted in Section 3.2, both observed and predicted values were converted into trend time series. By categorizing upward trends as positive and downward as negative, we established a confusion matrix, and utilizing this matrix, we calculated F1-score [37]. This focus on positive outcomes, while not utilised as a correlation metric in the variable selection process described in Section 3.2, offers critical insights as an evaluative measure of model outcomes. To eliminate the bias towards positive results, metrics were recalculated with inverted definitions of positive and negative. This allowed us to assess the model’s accuracy for both trend directions.

Moreover, we examined the alignment rate (accuracy) between the trend time series and Pearson’s product-moment correlation. This accuracy metric conveyed the congruence extent between observed and predicted value trends, facilitating an intuitive understanding. Nonetheless, as underscored in Section 3.2.1, evaluating upward and downward trends impartially posed a challenge when observed value trends were skewed. To circumvent this limitation, Pearson’s product-moment correlation for the trend time series was employed as an ancillary evaluative metric to ascertain the model’s overall balanced forecasting performance.

## 4. Results

### 4.1. Fixed-Period Model

#### 4.1.1. Application of Variable Selection

This section assesses the forecasting capability of the dependent variable using a fixed-period model (Section 3.7.1) within the context of the Greater Tokyo Area (encompassing Tokyo, Saitama, Chiba, Kanagawa, Ibaraki, Tochigi, Gunma, and Yamanashi). The learning period for the analysis spanned from 1 June 2020, to 31 May 2021. The validation period extended from 1 June 2021, through 12 October 2022, which marked the conclusion of the seventh wave. Variable selection for the linear regression model used the methodologies delineated in Section 2 and Section 3.

At first, 632 candidate independent variables, combining GPS and blog data, were considered. Following preprocessing procedures outlined in Section 2, which involved filtering out low-frequency words and terms with extreme peaks, 365 independent variables remained.

**Figure 7:**
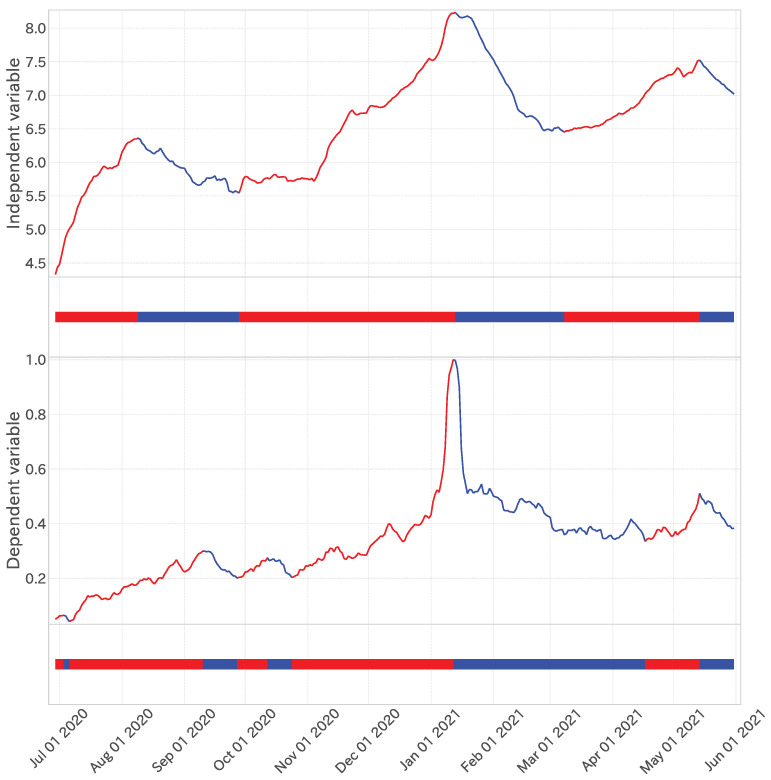
The first panel depicts the time series of the dependent variable after trend decomposition; red indicates an up trend, while blue indicates a down trend. The second panel shows the result of projecting the first panel onto one dimension. The third and fourth panels similarly illustrate the time series for the explanatory variable “禍” (disaster).

Each variable was then assigned a lag time, ranging from a minimum of 7 days to a maximum of 28 days. This resulted in a total of 8030 lagged time series variables. By applying the variable selection methodology to this assortment, we selected 330 variables based on their trend correlation with the dependent variable. In this phase, we excluded 35 variables from the original set of 365, focusing on the lag times that showed the strongest correlations. For example, the variable “禍” (disaster) exhibited its optimal lag at 9 days, as shown in Figure 7.

From this refined process, 94 variables were identified based on their Spearman rank correlation with the dependent variable. Notably, variables such as “禍” (disaster) with a 9-day lag exhibited substantial correlations (Table 1).

**Figure 8:**
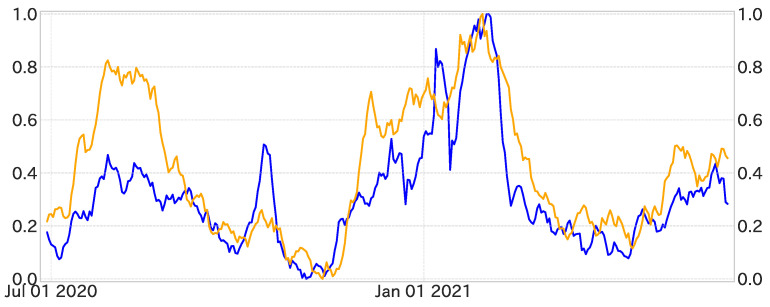
The blue time series represents “入院” (hospitalization), and the yellow time series represents “患者” (patients). These two series were grouped as they exhibited a Spearman’s rank correlation coefficient of 0.857. “入院” (Hospitalization) was correlated more strongly with the dependent variable, leading to the exclusion of “患者” (patients).

**Figure 9:**
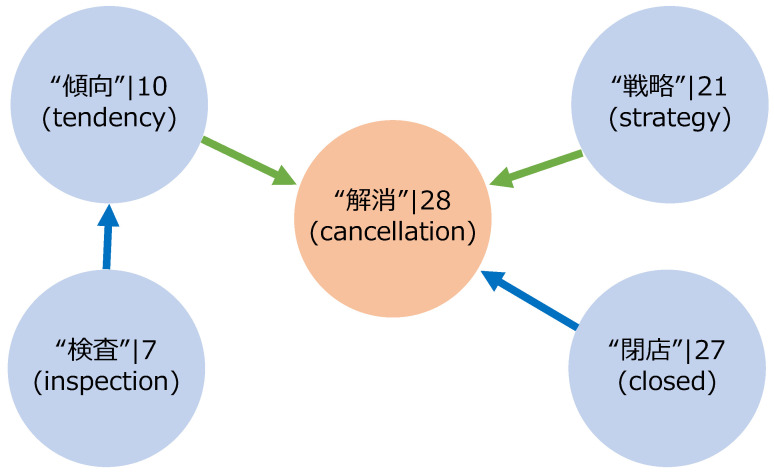
A part of the variable selection process in the fixed-period model is depicted in this figure. It shows the relationships between the final selected variables (red nodes) and those excluded through grouping and spurious correlations (blue nodes). The numbers in the diagram represent the best lag values determined during the correlation extraction phase. Blue arrows indicate that the variable at the arrow’s base is included in the variable group pointed to by the arrow’s head. In contrast, green arrows signify that the variable at the arrow’s base was determined to have a spurious correlation with the dependent variable due to the variable at the arrow’s head.

The subsequent grouping phase (Section 3.4) narrowed the field to 12 variables. During that process, terms related to “コロナ禍” (pandemic), “ワクチン” (vaccination), and “種” (variant) were associated with “禍” (disaster), while “患者” (patients), “死亡” (deaths), and “陽性” (positive) were linked to “入院” (hospitalization) (Figure 8). This step uncovered natural correlations driven by lexical similarity or contextual concurrence—the final selection (Section 3.5) comprised 7 variables, after identification and elimination of spurious correlations among the initial 12. As elucidated through this selection methodology, the intricate web of relationships between independent variables is aptly visualized for enhanced clarity in Figure 9.

Finally, a comparative analysis with the randomized time series discussed in Section 3.6 was undertaken. The analysis results unequivocally demonstrated that the adjusted coefficient of determination for the original series of independent variables consistently surpassed those for the randomized series. This finding underscored the model’s enhanced forecasting capability relative to the random walk model, substantiating the rationale for employing all selected variables.

The final assortment of independent variables comprised a 9-day lag series for “禍” (disaster), a 10-day lag series for “株” (strain),“ a 7-day lag series for “蔓延” (spread), a 7-day lag series for “入院” (hospitalization), a 12-day lag series for “影響” (impact), an 8-day lag series capturing the number of individuals in the “move” state from 18:00 to 24:00, and a 28-day lag series for “解消” (cancellation). With a minimal time lag of seven days, the linear regression model could forecast at least seven days in advance. For the meaning of these words in context, see Table A1.

#### 4.1.2. Linear Regression Analysis

In this section, we thoroughly examine the dependent variable’s forecasting performance using a linear regression model, incorporating the independent variables identified previously. The regression coefficients were estimated utilizing the least squares method.

**Figure 10:**
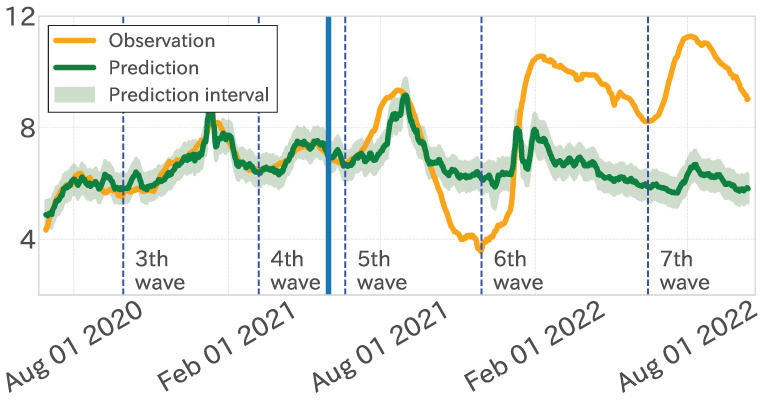
Results of the linear regression analysis for the fixed-period model. Observed values (yellow), model’s estimated values (green), and forecast interval (green band). The blue vertical lines distinguish between the learning and test periods.

First, the model’s fitting results during the learning period are scrutinized. The mean absolute error (MAE) was 0.189, signifying that the model effectively captured the observed values (refer to Figure 10 and Table 2 and Table 3). Nevertheless, the MAE value escalated to 2.504 over the entire test period, indicating a regression in performance efficacy. In contrast, the test period showed that the fifth wave was predicted with fairly good accuracy, as the position and height of the peaks were almost identical. After that, however, it was quite off (illustrated in Figure 10).

**Figure 11:**
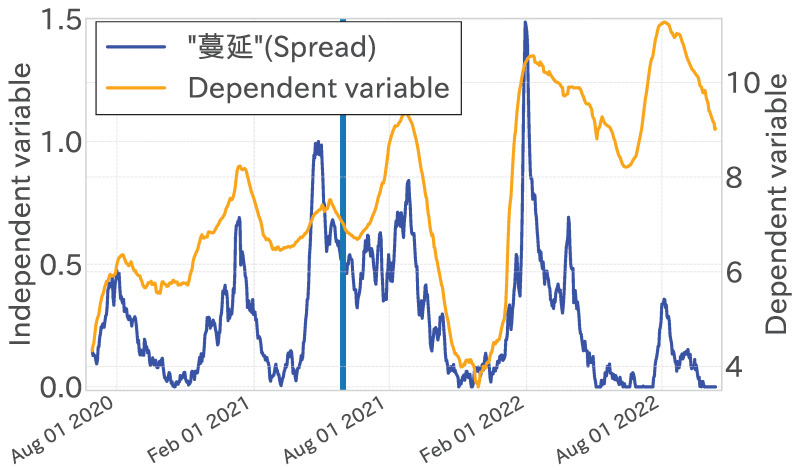
Comparison of the value obtained by multiplying the regression coefficient by the 7-day lag time series of “蔓延” (spread) (blue, right axis) with the observed values (yellow, left axis).

The analysis of the contribution of each independent variable within the linear regression model indicated that variables such as “禍” (disaster), “入院” (Hospitalization), and “蔓延” (spread) exhibited notably high regression coefficients, exerting a substantial influence on the predicted values. These variables demonstrated a robust correlation with the increases and decreases in the dependent variable’s time series, notably around peak timings, underscoring their critical importance. However, as the test period progressed, the influence of these variables decreased. This attenuation was linked to a diminished occurrence of the term “蔓延” (spread) after January 2022 (Figure 11). At the same time, we saw scenarios where the magnitude of the dependent variable increased while that of the independent variables decreased. Consequently, a degradation in regression efficacy and an elevation in MAE were discerned in the latter stages of the test period.

**Figure 12:**
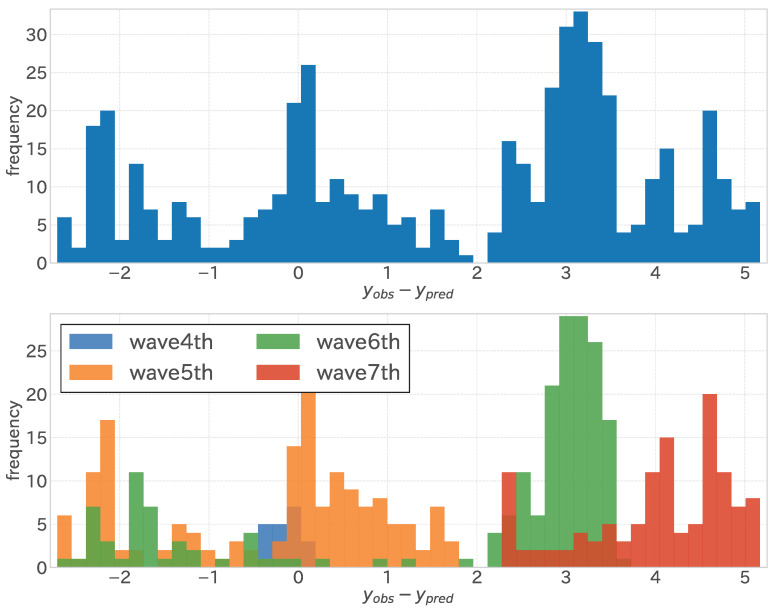
Histogram of errors (differences between observed and predicted values) for the fixed-period model during the test period. The upper section shows the entire test period, and the lower section shows each wave of infection.

Upon closer inspection of Figure 12, the distribution of errors in the linear regression analysis is highlighted. The depiction of the error distribution across the entire test period (illustrated at the top of Figure 12) indicates that it resembles the overlay of multiple distributions. This suggests the existence of diverse data point clusters, each characterized by a distinct error pattern. This observation becomes more pronounced when dissecting the error distribution corresponding to each infection wave (shown at the bottom of Figure 12), where it becomes apparent that the error dynamics identified in the earlier segment are attributable to variations across different data periods.

**Figure 13:**
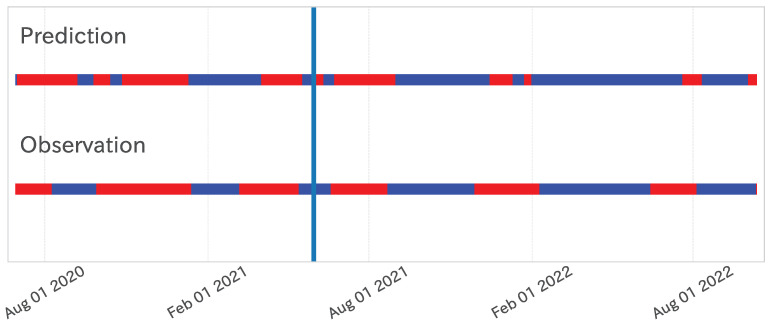
Results of decomposing the estimated values (**top**) and observed values (**bottom**) from the fixed-period model’s linear regression analysis into trend series. The red band represents the upward trend, and the blue band represents the downward trend. The blue vertical lines distinguish between the learning and test periods.

Next, we decomposed the observed and model-predicted values into trend time series to assess the capture of long-term trends through the matching rate (accuracy) and Pearson’s product-moment correlation. As Table 3 indicates, these metrics exhibited only minor variations between the learning and test periods. For example, the trend time series’ accuracy was 0.766 in the training period and 0.774 in the testing period, almost the same accuracy. Figure 10 and Figure 13 confirm that the model accurately forecast the timings of increases and decreases in observed values, maintaining a consistency similar to the learning period.

Furthermore, the forecasting accuracy for upward and downward trends was scrutinized. When categorizing the upward trend as positive, it was observed that the F1-score diminished during both the training and test periods. Conversely, the accuracy for the downward trend, considered positive, saw improvement during the test period. The proportions of observed upward versus downward trends between the training and test periods, as shown in Table 4, demonstrated a proportion reversal. This was attributed to temporal shifts in the spread of the COVID-19 infection and contraction rate.

Figure 13 illustrates lengthier phases of upward trends and brief downward trends during the learning period, signifying cycles of gradual infection spread followed by swift contractions. In contrast, the test period was characterized by swift infection surges succeeded by extended contractions. This alteration in the dependent variable’s mean and variance and the dynamics of infection spread contributed to the observed regression performance deterioration.

This section assessed the linear regression model’s efficacy across the learning and validation phases, elucidating the impact of divergent mean and variances between dependent and independent variables over time, the distribution pattern of errors by data period, and variations in the dependent variable’s fluctuations. Subsequent discussions introduce the sequential adaptive model to mitigate these identified challenges.

### 4.2. Sequential Adaptive Model with Infection Wave Segmentation

#### Results of the Sequential Adaptive Model

**Figure 14:**
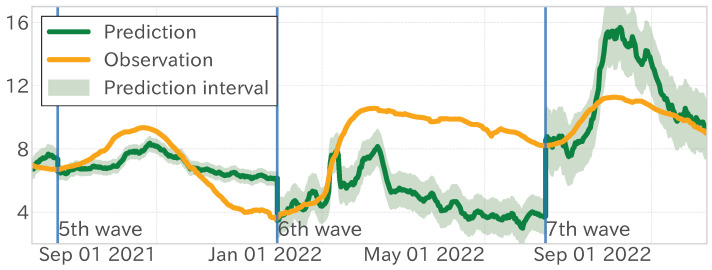
Results of linear regression analysis for the sequential adaptive model. Observational values (yellow) and the model’s forecasting values for each infection wave (green), with forecast intervals (green band). The blue vertical lines in the figure distinguish the infection waves determined by trend decomposition.

**Figure 15:**
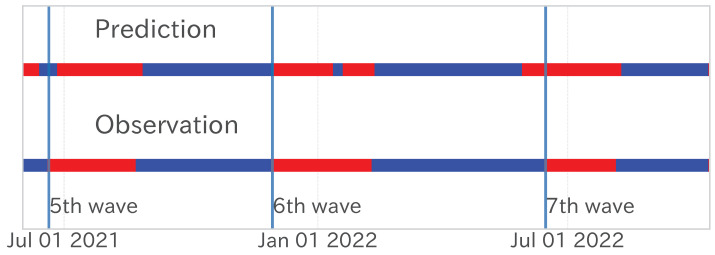
Results of the trend decomposition of the estimated values (**top**) and observed values (**bottom**) from the linear regression analysis of the sequential adaptive model. The red band represents an upward trend, and the blue band represents a downward trend. The blue vertical lines in the figure distinguish between the waves of infection determined by trend decomposition.

This section presents the outcomes of sequential learning according to our sequential adaptive model. We first examine the regression performance. As depicted in Figure 14 and Figure 15 and Table 5, there was a noticeable reduction in the mean absolute error (MAE) by approximately 10% relative to the fixed-period model, although regression errors persisted. A detailed examination of each infection wave’s results revealed that similar to the fixed-period model, mismatches in the time series’ mean and variance fluctuations between the dependent and independent variables negatively impacted regression efficacy. The forecast for the seventh wave tended to be overestimated compared to that of the other waves. This overestimation could be attributed to the variable “陽性” (positivity), which, as illustrated in Table 6, had the most substantial positive impact, while many other variables exhibited negative coefficients. During the validation phase, the frequency of “陽性” (positivity) remained consistent, whereas the prevalence of terms associated with negative coefficients diminished, leading to an overprediction. Furthermore, forecasts spanning the entire seventh wave—derived from data from the fifth and sixth waves—did not show a significant improvement over the random walk model in the final phase of variable selection.

Among the characteristics of the objective variable, a notable decrease in the number of new infections was observed during the New Year holidays of 2020, marking the fifth wave. Conversely, the sixth and seventh waves exhibited a trend in the mean and variance of new infections. The proposed regression analysis model did not include the increase in infection rates resulting from the emergence of highly transmissible COVID-19 variants (Omicron variant) as a dependent variable. This exclusion is considered one of the factors contributing to the observed discrepancies between the actual and predicted values.

**Figure 16:**
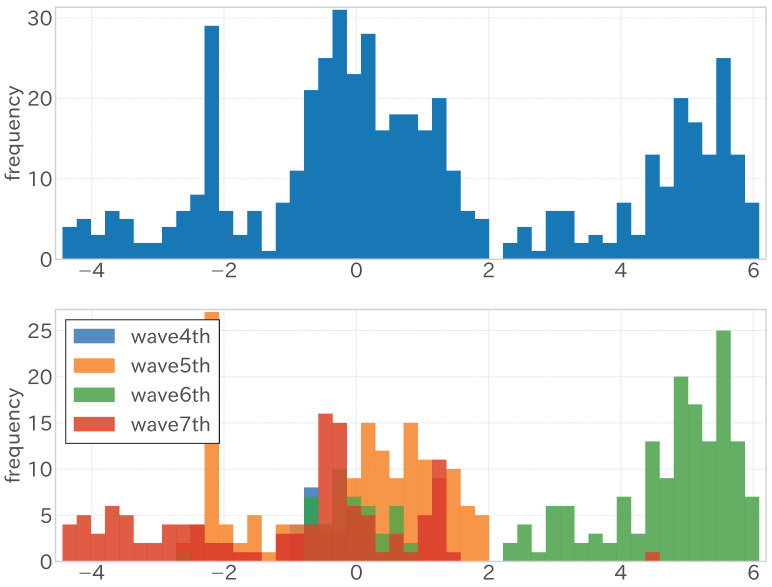
Histogram of errors (differences between observed and predicted values) for the sequential adaptive model during the test period. The upper section shows the entire test period, the lower section shows each wave of infection.

As shown in the upper and middle sections of Figure 16, the error distribution across infection waves exhibited variability, like in the fixed-period model. However, unlike the fixed-period model, where errors predominantly clustered around zero during the fifth wave, the sequential adaptive model demonstrated a distribution of errors around zero across each wave. As highlighted in the lower section of Figure 16, specific periods exhibited minor errors within each infection wave, while others presented more substantial discrepancies. Compared to the fixed-period model, where significant errors were prevalent across both periods, sequential learning in the sequential adaptive model enhanced data variability explanation across waves. Nevertheless, specific periods, such as the contraction phase of the sixth wave, displayed pronounced errors. The sixth wave saw a rapid surge in infections just before 2022, followed by a slow decline, failing to revert to pre-surge infection levels before the onset of the next wave. This unaccounted variability in the objective variable, absent from the training period, manifested here.

In the trend time series, when it comes to classification performance metrics such as accuracy and Pearson correlation coefficient (as shown in Table 5), it can be observed that the sequential adaptive model’s accuracy improved compared to the fixed-period model. The F1 scores for both the upward and downward trends showed a balanced improvement in both directions. It is worth noting that in the fixed-period model, the Recall for upward trends deteriorated due to changes in the variability of the objective variable. However, in the sequential adaptive model, the Recall values increased without worsening the precision values, indicating an improvement in accuracy.

Sequential learning is a technique that adapts to changes in COVID-19 infectivity and user behaviour by discarding old information and incorporating new data. Table 6 shows that the variables selected changed with each wave of infection, indicating changes in user behaviour. This emphasizes the importance of regular sequential learning.

Compared to the fixed-period model, the sequential adaptive model accurately and evenly forecast the long-term increase and decrease trends of time series. Additionally, it captured users’ changing interests across different waves of infection, making it superior in terms of result interpretation.

## 5. Discussion

### 5.1. Effects of Variable Selection

This section explores important considerations in constructing infectious disease forecast models using GPS and blog data, discussing the effectiveness of variable selection methods and the viability and limitations of sequential learning approaches using time series data.

For the variable selection, this study integrated multiple approaches, including trend correlation, Spearman’s rank correlation, grouping, removal of spurious correlations, and comparison with randomized time series. This process effectively balanced excluding irrelevant variables with selecting significant ones, leveraging the strengths of each method. Specifically, Spearman’s rank correlation captures the relatedness of time series data, but its limitations become apparent when the time series’ mean and variance fluctuate over time. For instance, variables whose mean and variance change due to unrelated factors over time might falsely exhibit high correlations when they are ranked. In our case, as time progressed, the mean and variance of the new infection count series increased, whereas a variable unrelatedly decreasing in mean and variance could easily show a negative correlation upon ranking. To circumvent this, the study initially utilised trend correlation of the time series. Although the new infection count series gradually increased in mean and variance, it fluctuated with expansions and contractions of infections. The study effectively excluded such variables by filtering variables that fluctuated in expansion and contraction periods using the trend series’ Pearson correlation coefficient.

Furthermore, analysing relationships among three variables, including the objective variable, by grouping variable correlations and removing spurious correlations, allowed for a deeper analysis. For example, Figure 9 shows that “閉店” (closed) had a similar variability to “解消” (cancellation). In addition, “戦略” (strategy) and “傾向” (tendency) were determined to be pseudo-correlations caused by “解消” (cancellation). This approach adapted to societal trends and shifts in the public interest, effectively capturing the objective variable’s long-term trends.

As shown in Section 4, by employing the variable selection methods proposed in this study, we were able to extract variables capable of forecasting the trends of infectious disease data. Section 4.1 demonstrated a decline in regression performance due to the mean and variance discrepancy between the objective and independent variables while maintaining stability in the trend correlation between the training and test periods. Section 4.2, although unable to fundamentally eliminate regression errors due to mean and variance discrepancies, demonstrated improvement in regression performance as errors for each infection wave moved closer to zero, as seen in Figure 16. Moreover, despite the change in variables selected for each wave of infection, the trend correlation remained stable throughout the test period, achieving an approximate 12% increase in accuracy compared to the fixed-period model.

This variable selection process also evaluated lagged time series, enforcing at least a 7-day lag to ensure the model predicted new infection counts at least seven days in advance. While this study focused on forecasting models, approaches like that of Yamada et al. [23], which estimate indicators with about a month’s real-time publication delay, can also be considered. Using the original time series without lag or information from 7 days ahead may be appropriate. If forecasts can be made before publication, one month later, the model would be deemed sufficiently valuable for evaluation.

To support reproducibility and facilitate further application of our method, we have released the core source code for the proposed modelling pipeline [38].

### 5.2. Validation and Limitations of Sequential Learning

In this study, we constructed a forecasting model for COVID-19 new case counts looking seven days ahead. However, as mentioned in Section 5.1, it is easy to extend the forecast period by altering the procedure for creating a lagged time series. This section briefly compares the classification performance of trend time series for models extended to forecast 14 and 21 days, thereby succinctly validating the efficacy of sequential learning and outlining its limitations.

From Table 7, it is evident that for all forecasting models, the sequential adaptive model outperformed the fixed-period model across evaluation metrics. Although accuracy decreased as the forecast horizon extended, sequential learning improved model accuracy beyond the 7-day forecast.

The limitations of sequential learning are also discussed. The improvement in regression performance through sequential learning is illustrated in Figure 12 and Figure 16. While the error distribution in the fixed-period model’s test period shows a separation across different waves of infection, in the sequential adaptive model’s test period, there is a noticeable shift in the distribution towards zero for each wave, highlighting the impact of sequential learning and its capability to address the mean and variance discrepancy between the target and independent variables. However, as observed in the shrinkage period of the sixth wave, current methods are ineffective against errors arising from other causes, necessitating further model development.

While the proposed modelling pipeline is designed to handle time series with nonstationarity and shifting correlations, its evaluation in this study was limited to COVID-19. This restriction was due to the nature of the GPS-based mobility dataset provided by Agoop Corp., which covers only Japan from March 2020 to March 2023, a period in which COVID-19 was the dominant infectious disease. The dataset does not include earlier influenza seasons or more recent periods in which other pathogens might have become dominant, thereby limiting the availability of suitable ground-truth data for evaluating model performance on alternative diseases. Nevertheless, the methodology itself remains generalizable in principle, and future applications could extend to other infectious diseases once appropriate mobility and behavioural datasets become available.

### 5.3. Future Challenges

The previous section discussed the limitations of sequential learning. However, the results from Section 4 also made the limitations of using GPS and blog data clear, as well as those of linear regression models. Specifically, blog data became an unstable factor in forecasting due to the increasing discrepancy over time between the means and variances of word frequency and new case counts. Conversely, while GPS data series seldom experienced extreme decreases, their long-term correlation with the target variable was less stable than that of blog data after the fifth wave into 2022. The normalized stay-at-home data showed an increasing trend in the number of people going out. Overcoming these challenges may require refining the granularity of GPS and blog data or incorporating past information on new case counts in a self-regressive manner, among other strategies involving additional data sources or more complex analytical methods.

To refine existing data granularity, municipalities could segment GPS data to create a more detailed time series of movement patterns, or more comprehensive information could be appended to stay data. The frequency of co-occurring words or more detailed reports based on blog post content could be utilised for blog data. However, since regression performance in blog data was significantly impacted by decreased word frequency, approaches that refine granularity could exacerbate the issue. Therefore, stabilizing the time series’ mean and variance using a synthetic series of variables grouped in the variable selection process is essential. Moreover, as mentioned in Section 5.2, additional data sources or model complexities are required to explain the nonlinear fluctuations in the target variable not covered by sequential learning. For instance, incorporating intervention variables such as variants and vaccine efficacy in sequential learning for each wave of infection, as discussed by Ozaki et al. [12], could be a viable approach.

## 6. Conclusions

This study constructed a time series forecast model for infectious diseases by integrating GPS and blog data. By combining different data sources and integrating multiple variable-selection methods, and by adapting a trend-decomposition-based Pearson (Matthews) correlation that linked binary up or down trends of dependent and independent series, we proposed a novel approach to capturing the fluctuations in contagious-disease time series.

At the heart of the research was the effective combination of variable-selection methods and the construction of a sequential learning model based on natural data segmentation using trend decomposition. Specifically, we applied the ϵ–τ procedure to both the outcome and each candidate predictor, then calculated their Pearson correlation on the decomposed trend labels, which represents an adaptation not previously reported in epidemiological studies. This pipeline achieved balanced variable selection that excluded irrelevant variables while retaining important ones, forecasting the trend of new infections with an accuracy of about 90%. The comparison between the fixed-period model and the sequential adaptive model confirmed that a wave-wise adaptive learning approach, tuned to infection waves, better accommodated mean and variance shifts in new cases than a fixed learning period.

The significance of this research lies in its practical exploration of forecasting methods for infectious diseases using GPS and blog data, thereby improving forecast accuracy in public-health contexts. Operationally, the seven-day lead time provided by the model can be embedded in the weekly situation reports already compiled by local health offices; when predicted incidence exceeds a preset threshold, staff may pre-position PCR-testing booths, adjust hospital-bed capacity and vaccination-clinic schedules, and issue targeted risk-communication messages to encourage early testing and isolation. At the same time, our experiments highlight how the nonstationarity of infectious-disease data and the linear modelling assumption restrict ultimate accuracy, delineating clear challenges for future work.

Future research should introduce nonlinear models and incorporate additional data sources to capture factors beyond the wave-specific mean and variance, thereby further enhancing forecasting accuracy. The fruitful next step will be to co-design early-warning thresholds with public-health stakeholders and retrospectively evaluate how timely interventions informed by our model could reduce caseloads and resource strain. Developing analytical methods that reflect the data characteristics and limitations identified here will open new possibilities for infectious-disease forecast.

Variable-selection techniques capable of extracting informative features from big data while addressing multicollinearity and spurious correlation are in high demand across scientific domains. By selecting causally relevant predictors, researchers can enhance the interpretability, reliability, and forecasting power of their models, leading to more robust insights and decision-making processes.

## Figures and Tables

**Table 1:** The *p*-value for the Spearman rank correlation coefficient, calculated between the dependent variable and other factors, was obtained from a sample spanning 337 days.

Variable	*p*-Value of Spearman’s r	Lag Days	Sign of Correlation Coefficient
“禍” (Disaster)	$2.389\times 10^{-111}$	9	Positive
“株” (Strain)	$1.670\times 10^{-41}$	10	Positive
“蔓延” (Spread)	$1.725\times 10^{-39}$	7	Positive
“入院” (Hospitalization)	$9.470\times 10^{-27}$	7	Positive
“影響” (Impact)	$1.993\times 10^{-23}$	12	Negative
Move *	$3.441\times 10^{-23}$	8	Negative
“解消” (Cancellation)	$2.159\times 10^{-16}$	28	Negative

* “Move” represents the number of people “on the move” in the 1 km mesh from 18:00 to 24:00.

**Table 2:** Regression coefficients for the fixed-period model, arranged in descending order of the absolute value of Spearman’s rank correlation with the dependent variable.

Variable	Regression Coefficient	Lag Days
“禍” (Disaster)	2.48	9
“株” (strain)	−0.09	10
“蔓延” (Spread)	0.95	7
“入院” (Hospitalization)	1.18	7
“影響” (Impact)	−0.63	12
Move *	0.15	8
“解消” (Cancellation)	−0.60	28
Constant	5.43	

* “Move” represents the number of people “on the move” in the 1 km mesh from 18:00 to 24:00.

**Table 3:** This table compares the regression performance and trend time series classification performance of the fixed-period model and the sequential adaptive model during the test period. The observed and predicted values are broken down into trend time series. The upward trend is considered as negative results (upward) and the downward trend is considered as positive results (downward). The accuracy and Pearson Correlation remain consistent irrespective of class definition.

Evaluation	Training Period	Test Period
MAE	2.360	2.012
Accuracy	0.766	0.774
Pearson Correlation	0.499	0.507
F1-score (Up)	0.813	0.663
F1-score (Down)	0.623	0.830

**Table 4:** Proportion of observed upward versus downward trends between the train and test periods.

Periods	Upward	Downward
2nd Wave	42.5%	57.5%
3rd Wave	33.4%	66.6%
4th Wave	35.0%	65.0%
5th Wave	60.5%	39.5%
6th Wave	63.1%	36.9%
7th Wave	56.3%	43.7%

**Table 5:** This table compares the regression performance and trend time series classification performance of the fixed-period model and the sequential adaptive model during the test period. The observed and predicted values are broken down into trend time series. The upward trend is considered as negative results (upward) and the downward trend is considered as positive results (downward). The accuracy and Pearson Correlation remain consistent irrespective of class definition.

Evaluation	Fixed-Periodmodel (Test Periods)	Sequential Adaptive Model (Test Periods)
MAE	2.360	2.012
Accuracy	0.774	0.890
Pearson Correlation	0.507	0.775
F1-score (Upward)	0.663	0.864
F1-score (Downward)	0.830	0.908

**Table 6:** Results of forecasting the variables of each infection wave. The parentheses show the number of lag days, and the second line in each cell displays the regression coefficient.

Prediction for 5th Wave	Prediction for 6th Wave	Prediction for 7th Wave
“PCR検査” (PCR Test) (7 days)	“コロナ” (COVID-19) (7 days)	“陽性” (Positive) (7 days)
1.25	5.44	6.92
“蔓延” (Spread) (7 days)	“懸念” (Concern ) (24 days)	Work * (7 days)
0.68	1.22	−2.38
“突入” (Breakthrough) (11 days)	“出かける” (Go out) (9 days)	“スタート” (Start) (28 days)
0.51	−0.29	−1.11
“禁止” (Ban) (7 days)	“提供” (Provide) (18 days)	“減少” (Decrease) (28 days)
−0.03	1.78	−4.40
“拡散” (Dissemination) (7 days)	“ビール” (Bear) (19 days)	“使える” (Usable) (24 days)
0.10	−1.30	1.70
“いける” (Can go) (12 days)		“株” (Strain) (11 days)
0.50		−2.65
“得る” (Obtain) (7 days)		“期間中” (During) (9 days)
0.10		1.70

* “Work” represents the square of the number of people at “work” within 1 km mesh from 18:00 to 24:00.

**Table 7:** Comparison of trend time series classification performance by accuracy. Results of the fixed-period model and the sequential adaptive model for 7-, 14-, and 21-day-ahead forecasts.

Test Period (Accuracy)	7 Day	14 Day	21 Day
**Fixed-period model**	0.774	0.705	0.657
**Sequential adaptive model**	0.890	0.784	0.752

## Data Availability

The GPS data and blog data used in this study were provided by Agoop Corp. and Hottolink, Inc., respectively. The GPS data are subject to usage restrictions and were used under license for the current study, and thus are not publicly available. The blog data are no longer accessible online because the “kuchikomi @ kakaricho” service has been discontinued. However, the data may be made available by the authors upon reasonable request and with permission from Agoop Corp. and Hottolink, Inc.

## Appendix A. Supplemental Information on Word Translations

**Table A1:** Japanese COVID-19-related words and their contextual meanings.

Japanese Word	English Translation	Context-Specific Meaning
禍 (Ka)	Disaster	Often used as part of the compound word “コロナ禍” (korona-ka), referring specifically to the COVID-19 pandemic rather than a general disaster.
株 (Kabu)	Strain	In the context of COVID-19, this refers to the different variants or strains of the SARS-CoV-2 virus.
蔓延 (Mannen)	Spread	Specifically refers to the spread or prevalence of the COVID-19 virus in the population.
入院 (Nyuin)	Hospitalization	Refers to the hospitalization of individuals due to COVID-19 infection.
影響 (Eikyō)	Impact	Describes the various impacts of the COVID-19 pandemic on society, economy, and individuals.
解消 (Kaisho)	Cancellation	In the context of COVID-19, this may refer to the lifting of restrictions, resolution of the pandemic, or the alleviation of its effects.

## Appendix B. Factors Contributing to Improvement with the Sequential Adaptive Model

In this study, we compared the fixed-period model (Section 4.1) and the sequential adaptive model (Section 4.2) and confirmed improvements in both regression performance and trend forecasting accuracy between the two models. The sequential adaptive model incorporated two major changes compared to the fixed-period model: the updating of features and regression coefficients for each wave of infections. However, these simultaneous updates made it challenging to determine which change contributed to the observed performance improvements.

To address this issue, we introduced the coefficient recalibration model, which only updated the regression coefficients from the fixed-period model, allowing for the separate evaluation of the effects of feature updates and regression coefficient updates. In the coefficient recalibration model, the features of the fixed-period model were maintained, while the regression coefficients were updated for each wave of infections.

The evaluation results are shown in Table A2, which provides the evaluation metrics for each model throughout the testing period. The fixed-period model used a one-year training period, whereas the coefficient recalibration model and the sequential adaptive model used training periods corresponding to two and three waves of infections, respectively.

The results indicate that both the coefficient recalibration model and the sequential adaptive model achieved improved mean absolute error (MAE) compared to the fixed-period model, with the regression performance of the coefficient recalibration model showing particularly significant improvement. This suggests that updating the regression coefficients plays an important role in improving the performance of the regression. However, in terms of trend forecasting accuracy, the coefficient recalibration model’s performance exhibited reduced performance when using a two-wave training period, but its performance surpassed that of the fixed-period model when using three waves. In contrast, the sequential adaptive model achieved improved accuracy even with a two-wave training period, demonstrating that feature updates strongly contribute to improving trend forecast accuracy.

These findings provide a deeper understanding of how updates to regression coefficients and features individually affect model performance.

**Table A2:** This table compares the regression performance and trend time series classification performance of the fixed-period model, the coefficient recalibration model, and the sequential adaptive model during the test period. The observed and predicted values are broken down into trend time series. The upward trend is considered as negative results (upward) and the downward trend is considered as positive results (downward). The accuracy and Pearson correlation remain consistent irrespective of class definition.　All values in the table denote the accuracy of the validation period. Each column also indicates the length of the study period.

Evaluation	Fixed-Period Model (1 Year)	Coefficient Recalibration Model (2 Wave)	Coefficient Recalibration Model (3 Wave)	Sequential Adaptive Model (2 Wave)	Sequential Adaptive Model (3 Wave)
MAE	2.360	2.123	2.092	2.012	2.001
Accuracy	0.774	0.645	0.792	0.890	0.892
Pearson Correlation	0.507	0.263	0.567	0.775	0.772
F1-score (Upward)	0.663	0.556	0.741	0.864	0.859
F1-score (Downward)	0.830	0.705	0.826	0.908	0.912
